# *In vitro *drug release behavior from a novel thermosensitive composite hydrogel based on Pluronic f127 and poly(ethylene glycol)-poly(ε-caprolactone)-poly(ethylene glycol) copolymer

**DOI:** 10.1186/1472-6750-9-8

**Published:** 2009-02-11

**Authors:** Chang Yang Gong, Shuai Shi, Peng Wei Dong, Xiu Ling Zheng, Shao Zhi Fu, Gang Guo, Jing Liang Yang, Yu Quan Wei, Zhi Yong Qian

**Affiliations:** 1 State Key Laboratory of Biotherapy, West China Hospital, West China Medical School, Sichuan University, Chengdu, 610041, PR China; 2 School of Life Science, Sichuan University, Chengdu, 610064, PR China

## Abstract

**Background:**

Most conventional methods for delivering chemotherapeutic agents fail to achieve therapeutic concentrations of drugs, despite reaching toxic systemic levels. Novel controlled drug delivery systems are designed to deliver drugs at predetermined rates for predefined periods at the target organ and overcome the shortcomings of conventional drug formulations therefore could diminish the side effects and improve the life quality of the patients. Thus, a suitable controlled drug delivery system is extremely important for chemotherapy.

**Results:**

A novel biodegradable thermosensitive composite hydrogel, based on poly(ethylene glycol)-poly(ε-caprolactone)-poly(ethylene glycol) (PEG-PCL-PEG, PECE) and Pluronic F127 copolymer, was successfully prepared in this work, which underwent thermosensitive sol-gel-sol transition. And it was flowing sol at ambient temperature but became non-flowing gel at body temperature. By varying the composition, sol-gel-sol transition and *in vitro *drug release behavior of the composite hydrogel could be adjusted. Cytotoxicity of the composite hydrogel was conducted by cell viability assay using human HEK293 cells. The 293 cell viability of composite hydrogel copolymers were yet higher than 71.4%, even when the input copolymers were 500 μg per well. Vitamin B_12 _(VB_12_), honokiol (HK), and bovine serum albumin (BSA) were used as model drugs to investigate the *in vitro *release behavior of hydrophilic small molecular drug, hydrophobic small molecular drug, and protein drug from the composite hydrogel respectively. All the above-mentioned drugs in this work could be released slowly from composite hydrogel in an extended period. Chemical composition of composite hydrogel, initial drug loading, and hydrogel concentration substantially affected the drug release behavior. The higher Pluronic F127 content, lower initial drug loading amount, or lower hydrogel concentration resulted in higher cumulative release rate.

**Conclusion:**

The results showed that composite hydrogel prepared in this paper were biocompatible with low cell cytotoxicity, and the drugs in this work could be released slowly from composite hydrogel in an extended period, which suggested that the composite hydrogel might have great potential applications in biomedical fields.

## Background

Cancer is a major public health problem in the world, which causes millions of death each year. Approximately 1.5 million new cancer cases and more than 500,000 deaths from cancer are projected to occur in 2008 in USA [1]. Now, one in four deaths in USA is due to cancer. As conventional therapy for cancer, chemotherapy has wide applications in clinical, which has been proven to be effective. Although chemotherapeutic agents may prolong the survival time of the patients, unfortunately most of them have severe side toxic effects, which would decline the life quality of patients. Most conventional methods for delivering chemotherapeutic agents, such as intravenous injection or oral ingestion, fail to achieve therapeutic concentrations of drugs, despite reaching toxic systemic levels. Novel controlled drug delivery systems (DDS) are designed to deliver drugs at predetermined rates for predefined periods at the target organ, which could be used to overcome the shortcomings of conventional drug formulations, therefore could diminish the side effects and improve the life quality of the patients [2,3]. Thus, a suitable controlled drug delivery system is extremely important for chemotherapy.

Hydrogels are a special class of macromolecules, which could absorb much water while maintaining their integrity in water. Over the past decades, the stimuli-sensitive hydrogel has attracted increasing attention owing to their responsiveness to the environmental stimulus, including chemical substances and changes in temperature, pH, or electric field [4-9]. The biodegradable thermosensitive physical crosslinked hydrogels have been extensively studied due to their great biodegradability, biocompatibility, and responsiveness to temperature. Therefore, biodegradable thermosensitive hydrogels have been investigated as *in situ *gel-forming system, such as controlled drug delivery, tissue repair, and cell encapsulation [10-20].

Poly(ethylene glycol)-poly(propylene glycol)-poly(ethylene glycol) triblock copolymer (PEG-PPG-PEG), known as Pluronic or Poloxamer, has been extensively studied as a potential drug delivery vehicle due to their excellent biocompatibility and thermosensitivity [21,22]. These copolymers have been widely used as emulsifiers, wetting agents, and solubilizers [23]. However, due to weak hydrophobicity of PPG block, the Pluronic F127 copolymer forms a fast-eroding gel and could persist a few hours *in vivo *at most, which greatly restricted its application as *in situ *gel-forming controlled drug delivery system.

In our previous study, we prepared a new kind of biodegradable and injectable thermosensitive poly(ethylene glycol)-poly(ε-caprolactone)-poly(ethylene glycol) (PEG-PCL-PEG, PECE) hydrogel controlled drug delivery system [24]. At low temperature, PECE hydrogel is injectable flowing sol, which could be easily mixed with pharmaceutical agent, and it forms non-flowing gel at body temperature as sustained drug delivery site *in vivo*. PCL and PEG are biocompatible and have been widely used in several FDA approved products [25-30]. PCL is lack of toxicity and has great permeability [31]. Due to combination of great advantages of PEG and PCL, the PECE hydrogel might have great potential application in biomedical field.

In our last work [32], we prepared a new biodegradable and injectable composite hydrogel based on PECE and Pluronic F127 copolymer. The composite hydrogel undergoes sol-gel-sol transition, which is free flowing sol at room temperature and becomes a non-flowing gel at body temperature. Our last work mainly focused on the synthesis, *in vivo *gel formation and degradation assay, and toxicity evaluation of the composite hydrogel. It is well known that Pluronic copolymer forms a fast-eroding hydrogel which could not persist longer than a few hours. And PECE hydrogel could persist 2 weeks in vivo [24]. According to our results, by simply altering the composition of PECE and Pluronic F127 copolymers, the *in vivo *sustained time of the composite hydrogel could be controlled, which could meet the different requirements of *in vivo *sustained time, therefore the composite hydrogel is very useful for its potential application as injectable *in situ *gel-forming drug delivery system.

In the present study, cytotoxicity and *in vitro *drug release behavior of the composite hydrogel were studied in detail. Many factors affecting *in vitro *drug release behavior of the composite hydrogel were investigated, including different kinds of drugs, initial drug loading, concentration of composite hydrogel, and chemical composition of the composite hydrogel. By altering the composition of composite hydrogel, sol-gel-sol transition behavior and *in vitro *drug release behavior of the prepared composite hydrogel could be controlled, which was of great importance for their further application as injectable *in situ *gel-forming drug release system.

## Methods

### Materials

Poly(ethylene glycol) methyl ether (MPEG, Mn = 550, Aldrich, USA), ε-Caprolactone (ε-CL, Alfa Aesar, USA), Pluronic F127 (Fluka, USA), Hexamethylene diisocyanate (HMDI, Aldrich, USA), Stannous octoate (Sn(Oct)_2_, Sigma, USA), Dulbecco's modified Eagle's medium (DMEM, Sigma, USA), 3-(4,5-dimethylthiazol-2-yl)-2,5-diphenyl tetrazolium bromide (MTT, Sigma, USA), bovine serum albumin (BSA, BR, BoAo Co. Ltd, China) and VB_12 _(Sigma, USA) were used without further purification. Honokiol (HK) were isolated and purified in our lab [33]. All the materials used in this article were analytic reagent (AR) grade and used as received.

### Preparation of composite hydrogel

PECE copolymer were synthesized and purified as reported previously [24]. Briefly, PEG-PCL diblock copolymers were prepared by ring opening polymerization of ε-CL initiated by MPEG using stannous octoate as catalyst; PEG-PCL-PEG triblock copolymers were synthesized by coupling PEG-PCL diblock copolymers using HMDI as coupling agent [18,24]. The just-obtained PECE block copolymers were dissolved in dichloromethane, and then reprecipitated from the filtrate using excess cold petroleum ether. Then, the mixture was filtered and vacuum dried to constant weight at room temperature. The purified copolymers were kept in air-tight bags before further use.

The obtained PECE copolymer was characterized by FTIR (NICOLET 200SXV, Nicolet, USA), ^1^H-NMR (Varian 400 spectrometer, Varian, USA), and GPC (Agilent 110 HPLC, USA). The M_n _and PEG/PCL ratio of PECE triblock copolymer calculated from ^1^H-NMR spectra was 3408 and 960/2448 respectively. Macromolecular weight and macromolecular weight distribution (polydispersity, PDI, M_w_/M_n_) of PECE triblock copolymer determined by GPC were 4391 and 1.30 respectively [24].

**Figure 1:**
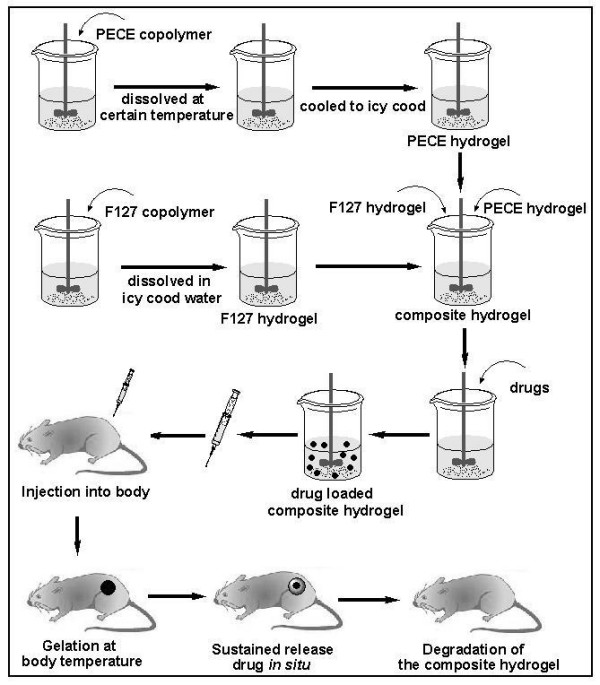
**Preparation scheme of demonstrated injectable thermosensitive composite hydrogel**. PECE hydrogel solutions were prepared by dissolving PECE copolymers in deionized water at a designated temperature then cooled to 4°C. Then, pluronic F127 were dissolved in icy cold deionized water to a transparent solution. Subsequently, the obtained two solutions were mixed together under mild agitation to obtain homogeneous liquid solution, and then drug were added into the composite hydrogel. The prepared hydrogel containing drug was inhaled into injector and injected into or around the focus of infection in animal. Thus, composite sol turned into gel state and acted as depots for sustained release of drug *in situ *when the cold sol is warmed to body temperature (37°C)*in vivo*. At last, for the degradation of the composite hydrogel, the introduced drug delivery system was gradually emanated from body.

Preparation scheme of composite hydrogel was described in Fig. 1. Aqueous PECE solutions were prepared by dissolving PECE copolymers in deionized water at a designated temperature then cooled to 4°C. Then, different amounts of Pluronic F127 were dissolved in icy cold deionized water to a transparent solution. Subsequently, the obtained two solutions were mixed together under mild agitation to obtain a homogeneous liquid solution. The final solution contained a given concentration and composition of the two copolymers to form the different composite hydrogel samples. The composite hydrogels prepared in this work were listed in Table 1.

**Table 1:** The composite hydrogels prepared in this work

Code	PECE : Pluronic F127 (w/w, %)	Phase transition	The concentration region with phase transition behavior (Wt%)
S1	100 : 0	Sol-gel-sol	15% to 35%
S2	80 : 20	Sol-gel-sol	25% to 35%
S3	60 : 40	Sol-gel-sol	20% to 35%
S4	40 : 60	Sol-gel-sol	20% to 35%
S5	20 : 80	Sol-gel-sol	20% to 35%
S6	0 : 100	Sol-gel-sol	15% to 35%

### Sol-gel-sol phase transition behavior study

Sol-gel-sol phase transition diagrams of composite hydrogels were recorded using test tube-inverting method [18,24]. The sol-gel-sol transition was visually observed by inverting the vials, and conditions of sol and gel were defined as "flowing" and "non-flowing" in one minute, respectively.

In this work, the volume of the composite hydrogel solution was kept to 0.5 mL in total, regardless of the concentration. After incubated in water bath at 4°C for 20 minutes, the hydrogel samples were slowly heated at a rate of 0.5°C/min, from 4°C to the temperature when the sol states occurred again.

### Cytotoxicity assay of composite hydrogel

Cytotoxicity evaluation of composite hydrogel (S1, S3, S4, and S6) was performed by 3- [4,5-dimethylthiazol-2-yl]-2,5-diphenyl tetrazolium bromide (MTT) assay using HEK293 cells, which were grown in DMEM with 10% of fetal bovine serum (FBS) and were cultured in a humidified atmosphere containing 5% CO_2 _at 37°C. Cell suspensions were distributed in a 96-well plate at a density of 1 × 10^4 ^cells/well in DMEM medium with 10% of FBS for 24 hours. Then, the medium were replaced by 200 μl of fresh DMEM per well containing different amounts of composite hydrogel copolymers (the amount of PECE and Pluronic F127 copolymer, which do not contain water) from 50 μg/ml to 2500 μg/ml, respectively. After 48 hours, the cell cultures were washed with PBS solution and MTT assay was conducted. Untreated cells were taken as control with 100% viability. The cell cytotoxicity of composite hydrogel was defined as the relative viability (%) which correlates with amount of liable cells compared with cell control.

### Preparation of honokiol micelles

Honokiol (HK) micelles were prepared and characterized in our previous work [34]. Briefly, certain amount of HK was dissolved into Et Ac to form HK solution. Then, the prepared HK-Et Ac solution was introduced into 4 ml of F127 aqueous solution at the concentration of 5%w/w under extreme stirring by T10 (T10, IKA, German). About 10 min later, oil in water (O/W) emulsion well formed. Then, Et Ac was evaporated in rotator evaporator ((BÜCHI, Switzerland) and the HK micelles were obtained. The content of HK in the prepared HK micelles suspension was determined by high-performance liquid chromatography (HPLC). At last, the HK micelles slurry was lyophilized and the power was stored at 4°C before further use.

### *In vitro *drug release behavior from the composite hydrogel

#### Release behavior of hydrophilic small molecular drugs

VB_12 _was used as model to determine the release behavior of hydrophilic small molecular drug from composite hydrogel *in vitro*. 200 μl of VB_12 _loaded composite hydrogel (30 wt% of S1 with 1 mg of VB_12_, 30 wt% of S4 with 1 mg of VB_12_, 30 wt% of S6 with 1 mg of VB_12_, 30 wt% of S3 with 1 mg of VB_12_, 30 wt% of S3 with 2 mg of VB_12_, 20 wt% of S3 with 1 mg of VB_12_, respectively) were placed into 4 mL-Eppendorf (EP) tubes and allowed to gel in an incubator at 37°C for 12 h. Then, the gels were immersed in 1 mL of PBS (pH = 7.4) and were shaken at 100 rpm at 37°C. At specific time intervals, all the release media were removed and replaced by fresh release media. After centrifuged at 13000 rpm for 10 min, the supernatant of the removed release media were collected and stored at -20°C until analysis. The collected supernatants were detected on UV spectrophotometer at 362 nm to determine the concentration of VB_12_. The accumulatively released VB_12 _was calculated according to the following equation [24]:

(1) *Q *= *C*_*n*_*V*_*t *_+ *V*_*s*_Σ*C*_*n*-1_

Where Q was accumulatively released weight, and C_n _was the VB_12 _concentration at time t. V_t _was the volume of medium (V_t _= 1 mL), and V_s _was the volume of solution removed from supernatant (V_s _= 1 mL).

#### Release behavior of hydrophobic small molecular drugs

Freshly prepared HK micelles loaded composite hydrogel were used to assay *in vitro *release behavior of hydrophobic small molecular drugs. In detail, 200 μl of prepared HK micelles loaded composite hydrogel (30 wt% of S1 with 1 mg of HK, 30 wt% of S3 with 1 mg of HK, 30 wt% of S6 with 1 mg of HK, 30 wt% of S4 with 1 mg of HK, 30 wt% of S4 with 2 mg of HK, 20 wt% of S4 with 1 mg of HK, respectively) were transferred into 4 mL-EP tubes and allowed to gel in an incubator at 37°C for 12 h. Then, the gels were immersed in 1 mL of PBS (pH = 7.4) and were shaken at 100 rpm at 37°C. At specific time intervals, all the release media were removed and replaced by fresh release media. After centrifuged at 13000 rpm for 10 min, the supernatant of the removed release media were collected and stored at -20°C until analysis.

The concentration of HK was determined by HPLC Instrument (Waters Alliance 2695). Solvent delivery system equipped with a column heater and a plus autosampler. Detection was taken on a Waters 2996 detector. Chromatographic separations were performed on a reversed phase C18 column (4.6 × 150 mm–5 um, Sunfire Analysis column). And the column temperature was kept at 28°C. Acetonitrile/water (60/40, v/v) was used as eluent at a flow rate of 1 mL/min. The standard curve equation is: H = 105000*X+4680 (H: The area of peak; X: the concentration of HK) and the correlation coefficient is 0.999994.

#### Release behavior of hydrophilic macromolecular protein drugs

*In vitro *release behavior of BSA, which was used as the model protein or peptide drug, from BSA loaded composite hydrogel was studied in detail. The procedure was similar to section 2.7.2, except that the initial drug loading amount were 4 mg and 8 mg, respectively. The amount of BSA present in the supernatant was determined by bicinchoninic acid (BCA) assay using BCA™ Protein Assay Kit (PIERCE, USA). The SDS-polyacrylamide gel electrophoretic (SDS-PAGE) analysis was used to assay the stability of BSA in the supernatant.

All the release study experiments were repeated three times. All data are expressed as the mean ± S.D.

### Scanning electron microscopy (SEM) of composite hydrogel

SEM was employed to investigate morphology of composite hydrogel before and after drug release. The composite hydrogels (before drug release test and 8 hours after drug released) were quickly frozen in liquid nitrogen and lyophilized at -45°C for 72 h. The composite hydrogels were sputtered with gold before observation. In this study, morphology of prepared composite hydrogels was examined on JEOL SEM (JSM-5900LV, JEOL, Japan).

## Results and discussion

### Synthesis of PECE copolymers

The synthesis of PECE triblock copolymers has been reported in our previous work [18,24]. Briefly, ring-opening copolymerization of ε-CL onto MPEG was performed to synthesis PEG-PCL diblock copolymers, and stannous octoate was used as catalyst. PEG-PCL diblock copolymers were then coupled using HMDI as coupling agent to produce the biodegradable PEG-PCL-PEG triblock copolymers.

### Temperature-dependent sol-gel-sol transition behavior

**Figure 2:**
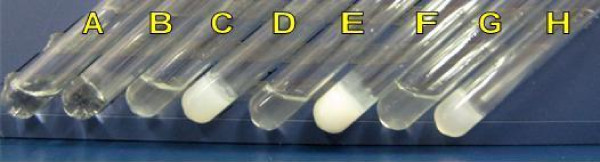
**Photograph of composite hydrogel (30 wt%) at different temperature**. S6 at 10°C (A) and 37°C (B); S4 at 10°C (C) and 37°C (D); S3 at 10°C (E) and 37°C (F); S1 at 10°C (G) and 37°C (H).

**Figure 3:**
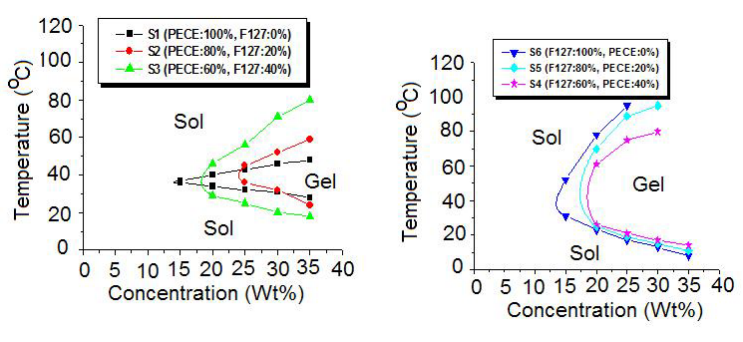
**Sol-gel-sol transition phase diagram of composite hydrogel**.

PECE and Pluronic F127 copolymers are both amphiphilic in nature, whose aqueous solution individually presented sol-gel-sol transition behavior. The composite hydrogel prepared in this work were composed of the two copolymers. As presented in Table 1, composite hydrogel based on PECE and Pluronic F127 copolymers from S1 to S6 all showed temperature-dependent reversible sol-gel-sol phase transition. The composite hydrogel flowed freely at lower temperature, but became a non-flowing gel at body temperature about 37°C (Fig. 2). Fig. 3 presented the sol-gel-sol phase transition diagrams of prepared composite hydrogel. When the copolymer concentrations are above the critical gelation concentration (CGC), aqueous solutions of composite hydrogel changed from "sol" phase to "gel" phase with increase in temperature to the lower critical gelation temperature (LCGT). With further increase of temperature to upper critical gelation temperature (UCGT), the sol phase occurs.

According to Fig. 3, pure PECE hydrogel (S1) and pure Pluronic F127 hydrgel (S6) both have a CGC of approximately 15 wt%, but S6 have a much wider gelation window than that of S1. The UCGT of S6 at the concentration of 30 wt% and 35% was not detected from 0°C to 100°C. The CGC of S2, S3, S4, and S5 were 25 wt%, 20 wt%, 20 wt%, and 20 wt%, respectively, which were much higher than that of two pure hydrogels. By mixing the two hydrogel together, the CGC of the composite hydrogel increased accordingly. CGC of S2 increased approximately 10 wt% than that of S1, whereas CGC of S5 increased approximately 5 wt% than that of S6. This phenomenon indicated that concerning CGC, the influence of Pluronic F127 hydrogel on PECE hydrogel was more dramatic than that of PECE hydrogel on Pluronic F127 hydrogel. As shown in Fig. 3, with increase in PECE hydrogel content in composite hydrogel, the UCGT decreased significantly, whereas the LCGT increased slightly. The UCGT of S6 hydrogel at concentration of 30 wt% and 35 wt% could not be detected from 0°C to 100°C, but the UCGT at concentration of 30 wt% and 35 wt% were detected in S5 and S3 hydrogel, respectively, due to increase in PECE content.

Therefore, it was obvious that sol-gel-sol transition behavior of composite hydrogel depended on the composition of the PECE and Pluronic F127 hydrogel. In fact, by altering the composition of composite hydrogel, the temperature range of sol-gel-sol phase transition could be broadened to a certain extent, which might be very useful for their further application as injectable *in situ *gel-forming drug delivery system.

### Cytotoxicity study of composite hydrogel copolymers

**Figure 4:**
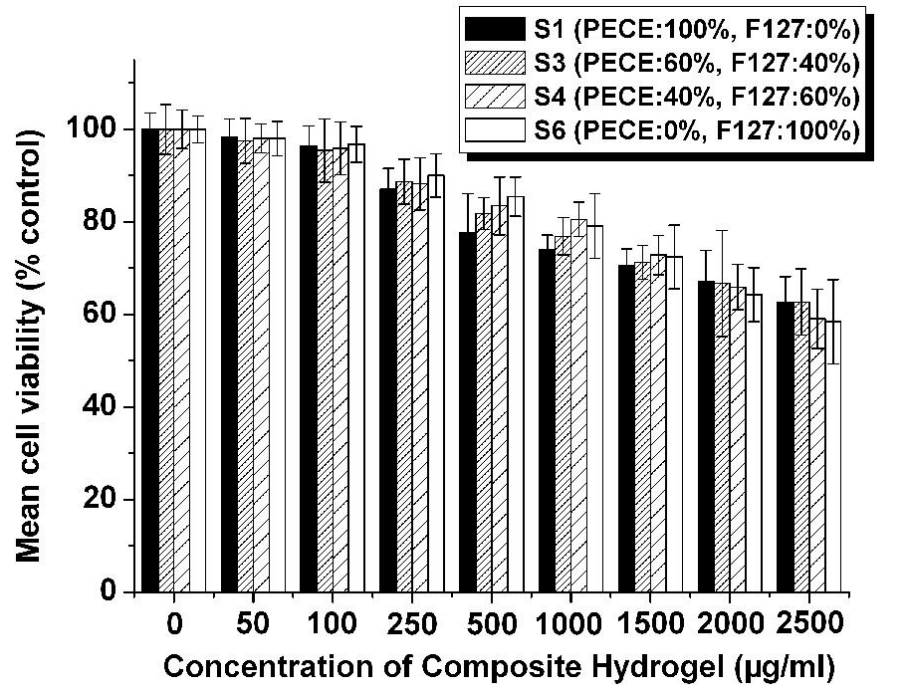
**HEK 293 cell viability assay**. Cell containing 1 × 10^4 ^cells in DMEM containing 10% FBS was incubated with S1, S3, S4 and S6 copolymers in 96-well in a humidified atmosphere containing 5% CO_2 _at 37°C for 48 h. Error bars represent the standard deviation (n = 6).

The cytotoxicity of the prepared composite hydrogels (S1, S3, S4, and S6) was evaluated by cell viability assay using HEK 293 cells. Fig. 4 exhibited the HEK 293 cell viability of composite hydrogel copolymer with different concentration gradient. As shown in Fig. 4, with increase of composite hydrogel copolymer amount, HEK 293 cell viability decreased accordingly. However, the 293 cell viability of S1, S3, S4 and S6 copolymers were yet higher than 72.5%, 76.6%, 78.0% and 71.4%, respectively, even when the input copolymers were 500 μg per well. According to Fig. 4, the HEK 293 cell viability of composite hydrogel copolymer decreased as increase of composite hydrogel copolymers. Compared with S3 and S4 copolymers group, S1 copolymer has higher cell viability at 20 μg/well or lower, but cell viability decreased significantly at 50 μg/well or higher. Cell viability study implied that the composite hydrogel copolymers prepared in this paper were biocompatible with low cell cytotoxicity. Therefore, composite hydrogel could be regarded as safe drug delivery carrier and is very promising for *in situ *gel-forming controlled drug delivery system.

### *In vitro *drug release profile of composite hydrogel

VB_12_, HK, and BSA were used as model drugs to investigate the release behavior of hydrophilic small molecular drug, hydrophobic small molecular drug, and protein drug from drug loaded composite hydrogels, respectively. Effect of the hydrogel composition, initial drug loading, and concentration of composite hydrogel on *in vitro *drug release behavior of the composite hydrogel were investigated in detail, which were discussed as follows.

### Release behavior of hydrophilic small molecular drug

**Figure 5:**
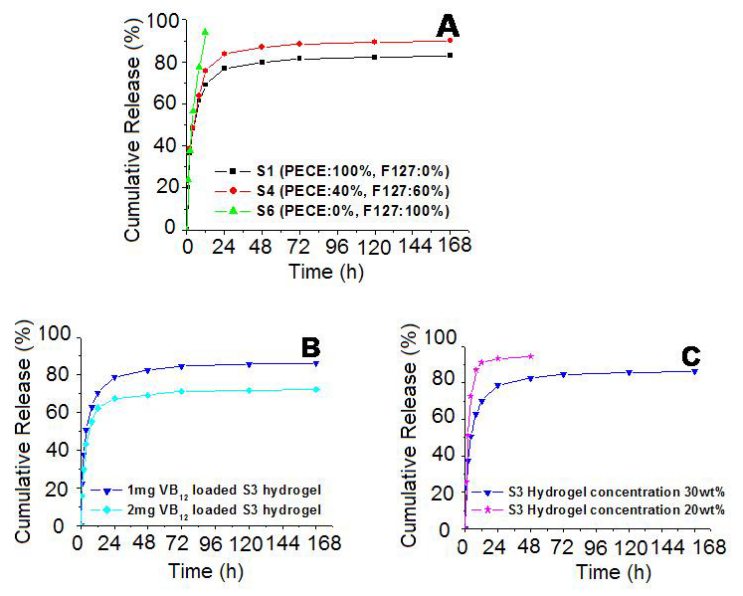
***In vitro *release behavior of VB_12 _from composite hydrogel**. A: release behavior of S1, S4, an S6 hydrogel with the same hydrogel concentration (30 wt%) and initial drug loading amount (1 mg). B: release behavior of 30 wt% S3 hydrogel with different initial drug loading amount (1 mg and 2 mg). C: release behavior of 1 mg VB_12 _loaded S3 hydrogel with different hydrogel concentration (20 wt% and 30 wt%). Error bars represent the standard deviation (n = 3).

*In vitro *release profile of VB_12 _from composite hydrogel in PBS was studied, and the results were shown in Fig. 5. According to Fig. 5, VB_12 _could be released in a sustained period. The hydrogel composition had great effect on VB_12 _release profile, and the results were shown in Fig. 5–A. S6 hydrogel disappeared completely in 12 hours with a cumulative release rate of approximately 94.2%, whereas S1 and S4 hydrogel could maintain their integrity in the whole release period. VB_12 _released faster and reached higher cumulative release rate (90.0%) from S3 hydrogel compared to S1 hydrogel (82.9%), which should be contributed to high composition (60 wt%) of fast-eroding Pluronic F127 in S3 hydrogel.

Effect of initial drug loading amount on release profile of S3 hydrogel was investigated. As shown in Fig. 5–B, S3 hydrogel containing twice amount of VB_12 _result in a significant decrease of cumulative release rate from 86.3% to 72.4%, which was constant to our previous work. With the same initial drug loading amount but lower hydrogel concentration (20 wt%) of S3, VB_12 _released faster and reached higher cumulative release rate (96.7%) compared to 30 wt% concentration S3 hydrogel (86.3%). Due to the higher composition of fast-eroding Pluronic F127 copolymer and lower hydrogel concentration, the 20 wt% S3 hydrogel was completely eroded in 48 hours. In 20 wt% hydrogel, an initial burst release of 25.6% of loaded VB_12 _occurred in the first one hour, followed by release of 94.7% in two days, whereas, in 30 wt% hydrogel, the cumulative release rate of one hour, two days and 7 days were 22.3%, 82.5%, and 86.3%, respectively.

### Release behavior of hydrophobic small molecular drug

HK, as multi-functional drug, have great potential application in human disease therapy, especially in cancer therapy. Previously, a rapid separation approach to isolate and purify HK had been developed using high-capacity high-speed counter-current chromatography (high-capacity HSCCC) by Chen et al in our lab [33]. For its great potential application and high hydrophobicity, HK was chosen for the hydrophobic model drug in this *in vitro *drug release study.

Due to high hydrophobicity, HK could not be well-disperse in the composite hydrogel to form homogeneous solution. HK micelles were employed to solve above-mentioned problem. The obtained HK micelles with average particle size of 33.34 nm and polydisperse index (PDI) of 0.036 could be well-dispersed in water, and it was stable. Only Pluronic F127, a composition of composite hydrogel, was remained in the HK micelles, which would not affect *in vitro *release behavior of composite hydrogel.

**Figure 6:**
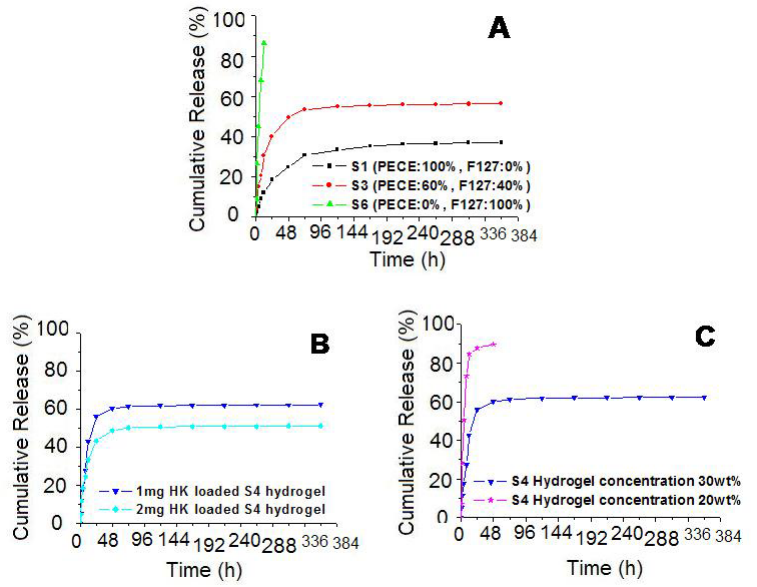
***In vitro *release behavior of HK from composite hydrogel**. A: release behavior of S1, S3, an S6 hydrogel with the same hydrogel concentration (30 wt%) and initial drug loading amount (1 mg). B: release behavior of 30 wt% S4 hydrogel with different initial drug loading amount (1 mg and 2 mg). C: release behavior of 1 mg HK loaded S4 hydrogel with different hydrogel concentration (20 wt% and 30 wt%). Error bars represent the standard deviation (n = 3).

The release behavior of HK from composite hydrogel was performed and the cumulative release profile was presented in Fig. 6. In Fig. 6–A, with increase in content of Pluronic F127 copolymer from 0% (S1) to 100% (S6), cumulative release rate and burst release rate (in one hour) increased from 37.1% to 86.5% and from 1.4% to 8.9%, respectively. According to Fig. 6–B, with increase of initial drug loading amount, the cumulative release rate of HK decreased dramatically from 62.1% to 51.0% in a 14-day period. As shown in Fig. 6–C, lower concentration of composite hydrogel led to higher cumulative release rate in a shorter time. Compared with VB_12_release profile, cumulative release rate and burst release rate of HK were much lower, which should be contributed to the high hydrophobicity of HK.

### Release behavior of hydrophilic macromolecular protein drugs

**Figure 7:**
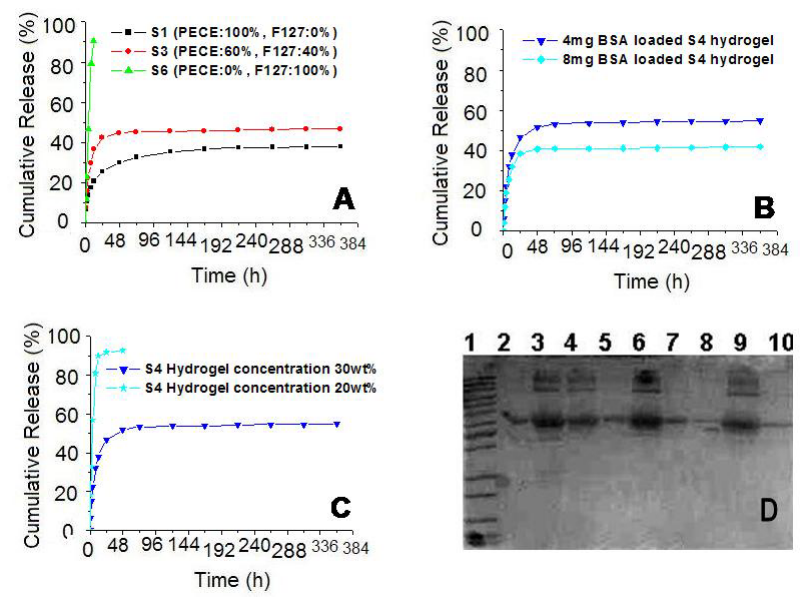
***In vitro *release behavior of BSA from composite hydrogel**. A: release behavior of S1, S3, an S6 hydrogel with the same hydrogel concentration (30 wt%) and initial drug loading amount (4 mg). B: release behavior of 30 wt% S4 hydrogel with different initial drug loading amount (4 mg and 8 mg). C: release behavior of 4 mg BSA loaded S4 hydrogel with different hydrogel concentration (20 wt% and 30 wt%). D: SDS-PAGE results of BSA *in vitro *release profile; Lane 1: marker; Lane 2: BSA standard; Lane 3: S1 at 24^th ^hour; Lane 4: S1 at 168^th ^hour; Lane 5: S1 at 360^th ^hour; Lane 6: S3 at 24^th ^hour; Lane 7: S3 at 168^th ^hour; Lane 8: S3 at 360^th ^hour; Lane 6: S3 at 12^th ^hour; Lane 10: S3 at 48^th ^hour. Error bars represent the standard deviation (n = 3).

*In vitro *release behavior of protein or peptide model drug from composite hydrogel was investigated, and the data were summarized in Fig. 7–A, B, and 7C. BSA could be released slowly from composite hydrogel in an extended period. As presented in Fig. 7–A, B, and 7C, effects of hydrogel composition, initial drug loading amount, and hydrogel concentration on BSA release profile were investigated in detail. The results were similar to the influence of these factors on HK release profile. SDS-PAGE was performed to evaluate the stability of BSA in the *in vitro *release period. According to Fig. 7–D, the major band for BSA appeared at about 67 KD (lane2 to lane10) according to the protein marker, which showed that BSA was stable in the experimental period.

Thus, composition of composite hydrogel, initial drug loading amount, and hydrogel concentration substantially affected the drug release behavior of composite hydrogel, where higher Pluronic F127 content, lower initial drug loading amount, or lower hydrogel concentration resulted in higher cumulative release rate, which means drug release rate of composite hydrogel could be controlled by simply altering the composition of PECE and Pluronic F127 copolymers. It is obvious that the influence of above-mentioned three factors is more dramatic on HK and BSA than VB_12_. Due to the great water solubility, VB_12 _was released very fast from the composite hydrogel, which could weaken the influence of the factors.

Besides the factors mentioned above, physical and chemical property of the drugs played an important role in their release behavior. According to Fig. 5 to Fig. 7, compared with hydrophobic small molecular drug, hydrophilic small molecular drug reached a higher cumulative release rate in a shorter period. In addition, cumulative release rate of hydrophilic small molecular drug was much higher than that of hydrophilic large molecular drugs.

### Morphology of composite hydrogel

**Figure 8:**
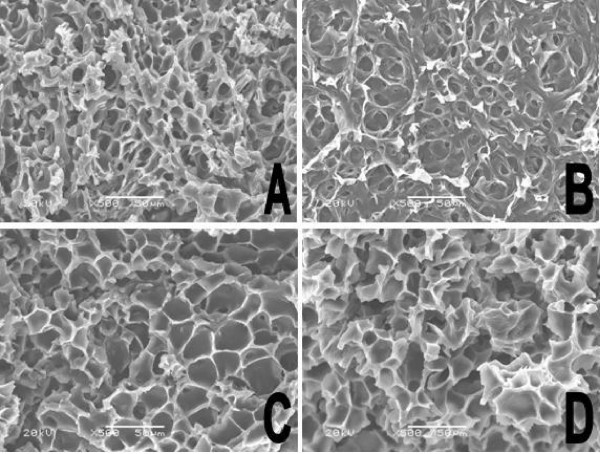
**SEM photograph of composite hydrogel before and after drug release**. S1 hydrogel before (A) and after (B) drug release for 8 hours. S3 hydrogel before (C) and after (D) drug release for 8 hours.

Interior morphology of composite hydrogel before and 8 hours after drug released was investigated by SEM. The composite hydrogels were frozen in liquid nitrogen and lyophilized for 72 h before the test. According to Fig. 8, all the hydrogel samples showed porous three-dimension structure, but the shape and mesh size of pores in the hydrogel were different. As shown in Fig. 8–A, S1 hydrogel before drug release presented approximately spherical pore with small mesh size. S3 hydrogel (Fig. 8–C), composed of 60% PECE hydrogel and 40% Pluronic F127 hydrogel, also showed spherical pores, but have larger mesh size compared to S1 hydrogel. The morphology of S1 and S3 hydrogel suggested that the composition of composite hydrogel have great influence on their interior structure, which dramatically affected the drug release behavior of composite hydrogel. Eight hours after drug released, S1 hydrogel could maintain its integrity, but the hydrogel surface eroded (Fig. 8–B). In Fig. 8–D, S3 hydrogel after immersed in PBS for 8 hours showed large pores and cracks, due to the fast-eroding of Pluronic F127 from the composite hydrogel.

## Conclusion

A series of novel biodegradable and thermosensitive composite hydrogel were successfully prepared in this work. The obtained composite hydrogel underwent sol-gel-sol transition with increase in temperature, which was flowing sol at ambient temperature and became non-flowing gel at body temperature. By varying the composition of PECE and Pluronic F127 copolymers, sol-gel-sol transition behavior and *in vitro *drug release profile of composite hydrogel could be adjusted, which was very useful for its potential applications as *in situ *gel-forming controlled drug delivery system.

## Abbreviations

PECE: poly(ε-caprolactone)-poly(ethylene glycol)-poly(ε-caprolactone) copolymer; PEG: poly(ethylene glycol); PCL: poly(ε-caprolactone); HPLC: high-performance liquid chromatography; DDS: controlled drug delivery systems; VB_12_: Vitamin B_12_; HK: honokiol,; BSA: bovine serum albumin; MPEG: Poly(ethylene glycol) methyl ether; F127: Pluronic F127, HMDI: Hexamethylene diisocyanate); Sn(Oct)_2_: Stannous octoate; DMEM: Dulbecco's modified Eagle's medium; MTT: 3-(4,5-dimethylthiazol-2-yl)-2,5-diphenyl tetrazolium bromide.

## Competing interests

The authors declare that they have no competing interests.

## Authors' contributions

QZY, WYQ, and GCY designed the experiments. And the research funds were supported by QZY and WYQ. GCY carried out experiments, analyzed the data, and wrote the manuscript; QZY corrected the manuscript. SS and YJL participated in the MTT cytotoxicity study of the hydrogels. ZXL participated in the in vitro release study from the composite hydrogels. DPW, GG and FSZ participated in synthesizing hydrogel and analyzing the data. All authors approved and read the final manuscript.
